# The genome sequence of the St Mark’s fly, *Bibio marci* (Linnaeus, 1758)

**DOI:** 10.12688/wellcomeopenres.17265.1

**Published:** 2021-10-22

**Authors:** Olga Sivell, Duncan Sivell, Natural History Museum Genome Acquisition Lab, Darwin Tree of Life Barcoding collective, Wellcome Sanger Institute Tree of Life programme, Wellcome Sanger Institute Scientific Operations: DNA Pipelines collective, Tree of Life Core Informatics collective, Darwin Tree of Life Consortium

**Affiliations:** 1 Natural History Museum, London, SW7 5BD, UK

**Keywords:** Bibio marci, St Mark’s fly, genome sequence, chromosomal

## Abstract

We present a genome assembly from an individual male *Bibio marci *(the St Mark’s fly; Arthropoda; Insecta; Diptera; Bibionidae). The genome sequence is 340 megabases in span. The complete assembly is scaffolded into six chromosomal pseudomolecules, with the X sex chromosome assembled.

## Species taxonomy

Eukaryota; Metazoa; Ecdysozoa; Arthropoda; Hexapoda; Insecta; Pterygota; Neoptera; Endopterygota; Diptera; Nematocera; Bibionoidea; Bibionidae; Bibio; *Bibio marci* (Linnaeus, 1758) (NCBI:txid189979).

## Introduction

*Bibio marci* (Diptera, Bibionidae), known as the St Mark’s fly, is a common and widely distributed species in Britain and Ireland. It can be found in grassland and at woodland edges, with a preference for lowland sites. The single flight period occurs in spring, from April to June in Britain and from May to June in Ireland ( Chandler, 2017; D’Arcy-Burt & Chandler, 1987; Freeman & Lane, 1985).

The males of *B. marci* swarm around hedges and high around trees, while females and mating pairs can be seen on vegetation ( D’Arcy-Burt & Chandler, 1987; Freeman & Lane, 1985). A gravid female digs up a cell in the soil, into which all of her eggs are oviposited in a single clutch. The female dies soon afterwards. The adult lifespan is short, likely less than a week ( Skartveit, 1997). The eggs hatch after approximately one month ( Freeman & Lane, 1985). The larvae of *B. marci* require humid conditions, are gregarious and can be found in the upper layers of soil, leaf litter, manure and in vegetable matter. They feed on decaying vegetation and the subterranean parts of living plants ( Freeman & Lane, 1985; Skartveit, 1997). Pupation occurs in cells dug out by larvae in the soil or rotten wood, with one pupa per cell ( Allen, 1975; Skartveit, 1997). This stage lasts about three weeks, after which the adult flies emerge ( Freeman & Lane, 1985).

Larvae of *B. marci* are occasional pests of grass, cereals, and other crops ( D’arcy-Burt & Blackshaw, 1991). They have been reported causing damage to celery, asparagus, rose, lettuce, grass (lawn), Saxifraga and Polyanthus in Britain ( Anderson, 1919; Edwards, 1925; Freeman & Lane, 1985), and potato tubers in Ireland ( Carpenter, 1920). *B. marci* larvae are an important food source for birds, particularly game birds ( Freeman & Lane, 1985; Parmenter, 1941).

The high-quality genome sequence described here is the first to be reported for *Bibio marci* and has been generated as part of the Darwin Tree of Life project. The sequence will aid understanding of the biology, physiology and ecology of the species.

## Genome sequence report

The genome was sequenced from a single male *B. marci* collected from Wigmore Park, Wigmore, Luton, England (latitude 51.88378, longitude -0.36861422). A total of 53-fold coverage in Pacific Biosciences single-molecule long reads and 127-fold coverage in 10X Genomics read clouds were generated. Primary assembly contigs were scaffolded with chromosome conformation Hi-C data. Manual assembly curation corrected 14 missing/misjoins, reducing the scaffold number by 57.14%.

**Figure 1.**
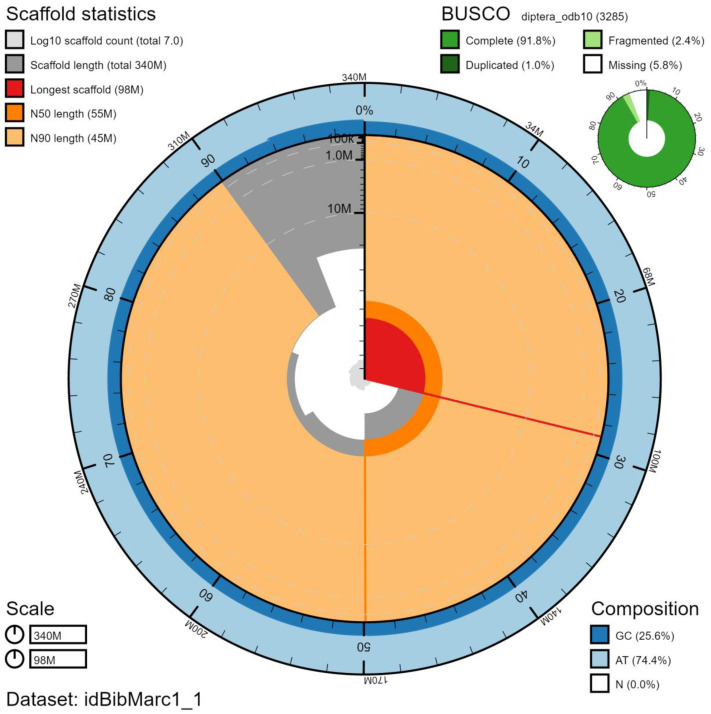
Genome assembly of *Bibio marci*, idBibMarc1.1: metrics. The BlobToolKit Snailplot shows N50 metrics and BUSCO gene completeness. The main plot is divided into 1,000 size-ordered bins around the circumference with each bin representing 0.1% of the 340,022,626 bp assembly. The distribution of chromosome lengths is shown in dark grey with the plot radius scaled to the longest chromosome present in the assembly (97,962,292 bp, shown in red). Orange and pale-orange arcs show the N50 and N90 chromosome lengths (54,570,542 and 45,408,480 bp), respectively. The pale grey spiral shows the cumulative chromosome count on a log scale with white scale lines showing successive orders of magnitude. The blue and pale-blue area around the outside of the plot shows the distribution of GC, AT and N percentages in the same bins as the inner plot. A summary of complete, fragmented, duplicated and missing BUSCO genes in the diptera_odb10 set is shown in the top right. An interactive version of this figure is available at https://blobtoolkit.genomehubs.org/view/idBibMarc1.1/dataset/idBibMarc1_1/snail.

The final assembly has a total length of 340 Mb in six sequence scaffolds with a scaffold N50 of 54.6 Mb ( Table 1). The complete assembly sequence was assigned to chromosomal-level scaffolds, representing five autosomes (numbered by sequence length) and the X sex chromosome ( Figure 1– Figure 4; Table 2). *B. marci* has an unknown sex chromosome system and no Y chromosome was recovered, despite the fact that the specimen was identified as male. However, there is no strong evidence to indicate that X and Y have been incorrectly merged together: the X chromosome has good contiguity and there is no evidence of misassembly. There is also good Hi-C linking across scaffold gaps. The mitochondrial genome was also assembled and is 13.2 kb in length.

**Table 1.** Genome data for *Bibio marci*, idBibMarc1.1.

*Project accession data*	
Assembly identifier	idBibMarc1.1
Species	*Bibio marci*
Specimen	idBibMarc1
NCBI taxonomy ID	219539
BioProject	PRJEB45122
BioSample ID	SAMEA7524263
Isolate information	Male, abdomen
*Raw data accessions*	
PacificBiosciences SEQUEL II	ERR6412035
10X Genomics Illumina	ERR6054781-ERR6054784
Hi-C Illumina	ERR6054785
*Genome assembly*	
Assembly accession	GCA_910594885.1
*Accession of alternate haplotype*	GCA_910594895.1
Span (Mb)	340
Number of contigs	25
Contig N50 length (Mb)	44.9
Number of scaffolds	7
Scaffold N50 length (Mb)	54.6
Longest scaffold (Mb)	98.0
BUSCO * genome score	C:91.8%[S:90.7%,D:1.0%], F:2.4%,M:5.8%,n:3285

*BUSCO scores based on the diptera_odb10 BUSCO set using v5.1.2. C= complete [S= single copy, D=duplicated], F=fragmented, M=missing, n=number of orthologues in comparison. A full set of BUSCO scores is available at https://blobtoolkit.genomehubs.org/view/idBibMarc1.1/dataset/idBibMarc1_1/busco

**Figure 2.**
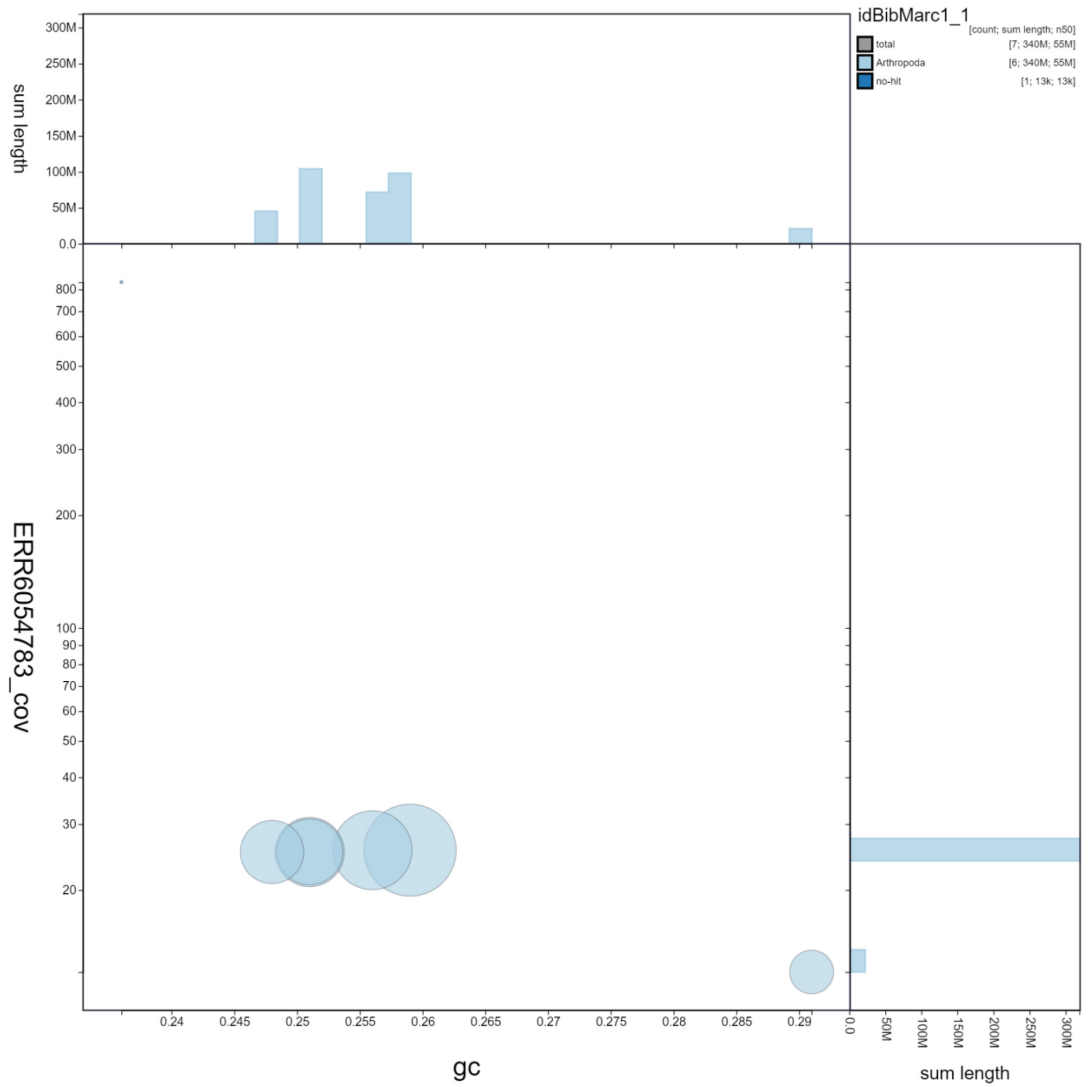
Genome assembly of *Bibio marci*, idBibMarc1.1: GC coverage. BlobToolKit GC-coverage plot. Chromosomes are coloured by phylum. Circles are sized in proportion to chromosome length. Histograms show the distribution of chromosome length sum along each axis. An interactive version of this figure is available at https://blobtoolkit.genomehubs.org/view/idBibMarc1.1/dataset/idBibMarc1_1/blob.

**Figure 3.**
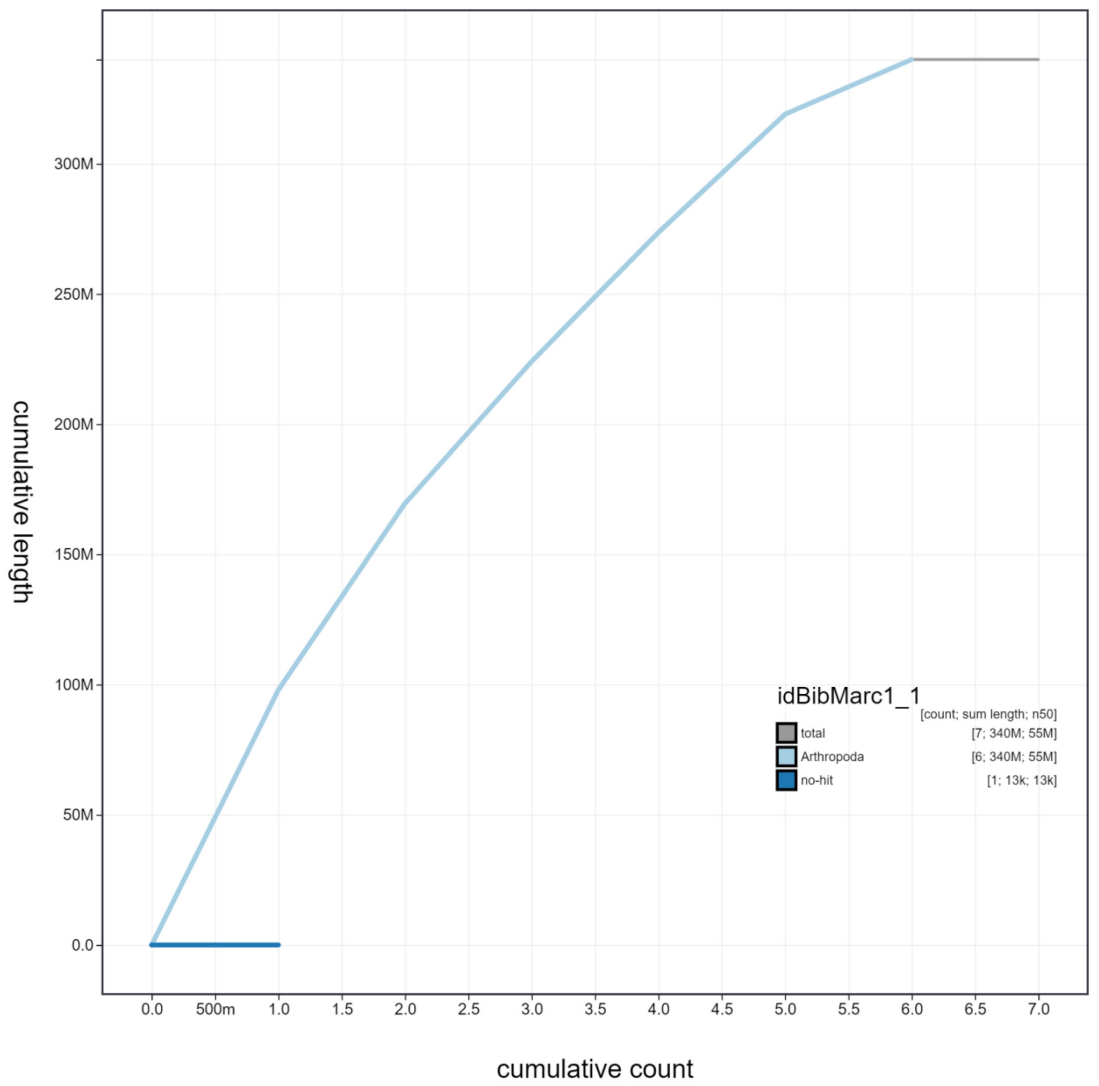
Genome assembly of *Bibio marci*, idBibMarc1.1: cumulative sequence. BlobToolKit cumulative sequence plot. The grey line shows cumulative length for all scaffolds. Coloured lines show cumulative lengths of scaffolds assigned to each phylum using the buscogenes taxrule. An interactive version of this figure is available at https://blobtoolkit.genomehubs.org/view/idBibMarc1.1/dataset/idBibMarc1_1/cumulative.

**Figure 4.**
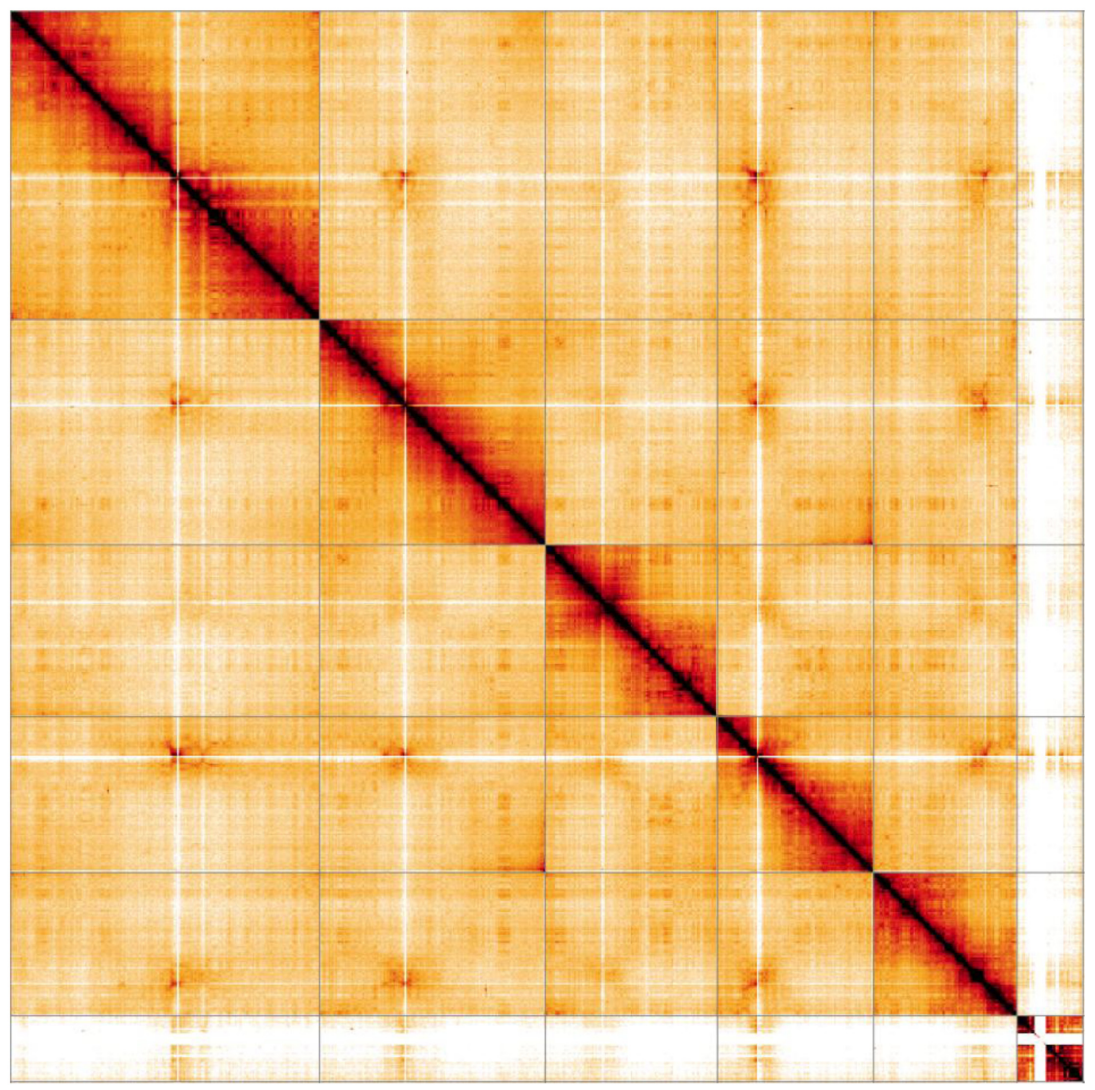
Genome assembly of *Bibio marci*, idBibMarc1.1: Hi-C contact map. Hi-C contact map of the idBibMarc1.1 assembly, visualised in HiGlass. Chromosomes are arranged by size from left to right and top to bottom.

**Table 2.** Chromosomal pseudomolecules in the genome assembly of *Bibio marci*, idBibMarc1.1.

INSDC accession	Chromosome	Size (Mb)	GC%
OU343114.1	1	97.96	25.9
OU343115.1	2	71.6	25.6
OU343116.1	3	54.57	25.1
OU343117.1	4	49.48	25.1
OU343118.1	5	45.41	24.8
OU343119.1	X	20.99	29.1
OU343120.1	MT	0.01	24.2

The assembly has a BUSCO v5.1.2 ( Simão *et al.*, 2015) completeness of 91.8% (single 90.7%, duplicated 10%, fragmented 2.4%, missing 5.8%) using the diptera_odb10 reference set. However, it is likely this relatively low BUSCO score does not reflect incompleteness of the genome assembly; analysis using the class-level insecta_odb10 lineage shows a completeness of 98.5% (single 96.7%, duplicated 1.8%, fragmented 0.6%, missing 0.9%) and other lineages have similarly high values. As such, we believe that the low score for the order-level lineage reflects divergence of *B. marci* from other Diptera. See here for a full set of BUSCO scores using all available lineages for this assembly. While not fully phased, the assembly deposited is of one haplotype. Contigs corresponding to the second haplotype have also been deposited.

## Methods

A single male *B. marci* was collected from Wigmore Park, Wigmore, Luton, England (latitude 51.88378, longitude -0.36861422) on 6 May 2020 by Olga Sivell, Natural History Museum, London, using a net. The morphological identification was provided by Duncan Sivell, Natural History Museum, London, based on ( Freeman & Lane, 1985). The sample was snap-frozen using dry ice and stored in a CoolRack. The sample was collected during a Covid-19 lockdown and owing to logistical issues, no image of the sample was taken.

DNA was extracted at the Tree of Life laboratory, Wellcome Sanger Institute (WSI). The ilGlaAlex1 sample was weighed and dissected on dry ice with tissue set aside for Hi-C sequencing. Tissue from the abdomen was disrupted to a fine powder using a powermasher. Fragment size analysis of 0.01–0.5 ng of DNA was then performed using an Agilent FemtoPulse. High molecular weight (HMW) DNA was extracted using the Qiagen MagAttract HMW DNA extraction kit. Low molecular weight DNA was removed from a 200-ng aliquot of extracted DNA using 0.8X AMpure XP purification kit prior to 10X Chromium sequencing; a minimum of 50 ng DNA was submitted for 10X sequencing. HMW DNA was sheared into an average fragment size between 12–20 kb in a Megaruptor 3 system with speed setting 30. Sheared DNA was purified by solid-phase reversible immobilisation using AMPure PB beads with a 1.8X ratio of beads to sample to remove the shorter fragments and concentrate the DNA sample. The concentration of the sheared and purified DNA was assessed using a Nanodrop spectrophotometer and Qubit Fluorometer and Qubit dsDNA High Sensitivity Assay kit. Fragment size distribution was evaluated by running the sample on the FemtoPulse system.

Pacific Biosciences HiFi circular consensus and 10X Genomics read cloud DNA sequencing libraries were constructed according to the manufacturers’ instructions. DNA sequencing was performed by the Scientific Operations core at the WSI on Pacific Biosciences SEQUEL II and Illumina HiSeq X instruments. Hi-C data were generated from abdomen tissue in the WSI Tree of Life Laboratory using the Arima v2.0 kit and sequenced in the Scientific Operations core on an Illumina NovaSeq 6000 instrument.

Assembly was carried out with Hifiasm ( Cheng *et al.*, 2021); haplotypic duplication was identified and removed with purge_dups ( Guan *et al.*, 2020). One round of polishing was performed by aligning 10X Genomics read data to the assembly with longranger align, calling variants with freebayes ( Garrison & Marth, 2012). The assembly was then scaffolded with Hi-C data ( Rao *et al.*, 2014) using SALSA2 ( Ghurye *et al.*, 2019). The assembly was checked for contamination and corrected using the gEVAL system ( Chow *et al.*, 2016) as described previously ( Howe *et al.*, 2021). Manual curation was performed using gEVAL, HiGlass ( Kerpedjiev *et al.*, 2018) and Pretext. The mitochondrial genome was assembled using MitoHiFi ( Uliano-Silva *et al.*, 2021). The genome was analysed and BUSCO scores generated within the BlobToolKit environment ( Challis *et al.*, 2020). Table 3 contains a list of all software tool versions used, where appropriate.

**Table 3.** Software tools used.

Software tool	Version	Source
Hifiasm	0.12	Cheng *et al.*, 2021
purge_dups	1.2.3	Guan *et al.*, 2020
SALSA2	2.2	Ghurye *et al.*, 2019
longranger align	2.2.2	https://support.10xgenomics.com/genome-exome/ software/pipelines/latest/advanced/other-pipelines
freebayes	1.3.1-17-gaa2ace8	Garrison & Marth, 2012
MitoHiFi	1	Uliano-Silva *et al.*, 2021
gEVAL	N/A	Chow *et al.*, 2016
PretextView	0.2.x	https://github.com/wtsi-hpag/PretextView
HiGlass	1.11.6	Kerpedjiev *et al.*, 2018
BlobToolKit	2.6.2	Challis *et al.*, 2020

The materials that have contributed to this genome note have been supplied by a Darwin Tree of Life Partner. The submission of materials by a Darwin Tree of Life Partner is subject to the Darwin Tree of Life Project Sampling Code of Practice. By agreeing with and signing up to the Sampling Code of Practice, the Darwin Tree of Life Partner agrees they will meet the legal and ethical requirements and standards set out within this document in respect of all samples acquired for, and supplied to, the Darwin Tree of Life Project. Each transfer of samples is further undertaken according to a Research Collaboration Agreement or Material Transfer Agreement entered into by the Darwin Tree of Life Partner, Genome Research Limited (operating as the WSI), and in some circumstances other Darwin Tree of Life collaborators.

## Data availability

European Nucleotide Archive: Bibio marci (St. Mark's fly). Accession number PRJEB45122; https://identifiers.org/ena.embl/PRJEB45122.

The genome sequence is released openly for reuse. The *B. marci* genome sequencing initiative is part of the Darwin Tree of Life (DToL) project. All raw sequence data and the assembly have been deposited in INSDC databases. Raw data and assembly accession identifiers are reported in Table 1. The voucher specimen has been accessioned at the Natural History Museum, London, under accession number NHMUK014111013.
