# Supplementary material for: The genome sequence of the St Mark’s fly, Bibio marci (Linnaeus, 1758)
Source: Wellcome Open Res. 2021 Oct 22;6:285. [Version 1] doi: 10.12688/wellcomeopenres.17265.1 (PMC9073268; doi:10.12688/wellcomeopenres.17265.1)
Supplement: Peer review history

# Peer review: Reviewer response for version 1

**DOI:** 10.21956/wellcomeopenres.19083.r50124

**Published:** 2022-05-05

**Authors:** Craig Wilding

**Affiliations:** 1 School of Biological and Environmental Sciences, Liverpool John Moores University, Liverpool, UK

This Genome Note provides a highly readable and clear summary of the genome assembly and annotation efforts for *Bibio marci*. It will be of interest to those studying this species, those interested in the wider evolutionary biology of insects, and potentially those interested in sex-determining mechanisms.

 Whilst the genome data (and very clear methods) are reported, unusually for DToL datasets there is no evidence of an RNASeq dataset – is this an oversight or has none been generated?

 I would be interesting in seeing further details of the manual curation that led to the welcome improvement in scaffold number and scaffold N50. This is alluded to but not fully explained.

 I note a discrepancy in the reported number of scaffolds – six (five autosomes and 1 X-chromosome) are stated in the text, but seven scaffolds are reported in Table 1 (though N _50_ values are the same in the text and Table).

 This assembly has a relatively low 91.8% complete BUSCO completeness. While the authors suggest that this is a consequence of using the diptera gene set (with >98% completeness when the insecta gene set is utilised *) *it is interesting to note that 96.6% completeness is seen for the drone fly *Eristalis tenax* (Hawkes *et al.* (2021 ^1^)) using the diptera gene set. As further insect genomes are generated this might provide further insight into whether this is an outlier or not.

Are sufficient details of methods and materials provided to allow replication by others?

Yes

Is the rationale for creating the dataset(s) clearly described?

Yes

Are the datasets clearly presented in a useable and accessible format?

Yes

Are the protocols appropriate and is the work technically sound?

Yes

Reviewer Expertise:

Genomics (invertebrates)

I confirm that I have read this submission and believe that I have an appropriate level of expertise to confirm that it is of an acceptable scientific standard.

## References

[1] : The genome sequence of the drone fly, Eristalis tenax (Linnaeus, 1758). *Wellcome Open Research* .2021;6: 10.12688/wellcomeopenres.17357.1 10.12688/wellcomeopenres.17357.1 34796273

# Peer review: Reviewer response for version 1

**DOI:** 10.21956/wellcomeopenres.19083.r48111

**Published:** 2022-01-19

**Authors:** Darren Obbard

**Affiliations:** 1 Institute of Evolutionary Biology, University of Edinburgh, Edinburgh, UK

This data note reports the sequencing and assembly of the genome of *Bibio marci* as part of the “Darwin Tree of Life” programme. In common with other data notes from this research effort, the reporting is standardised and quite brief. As such, I have very few comments to make.

 The approach is state-of-the-art, the raw data appear to be of a suitably high quality, and the assembly methods are appropriate. The provision of a voucher specimen is excellent. The public availability of raw data and genome assembly is excellent. The resulting genome is likely to be of very high quality, and I have no doubt that it will be of great value to any researchers working on this group of lies, or on the comparative or evolutionary genomics of insects more generally. The writing is clear, concise, and very easy to read. The authors have also managed to include sufficient allusion to the biology and research context of this fly to position the data note within the larger research literature.

 Below, I note a few inconsistencies or points that could usefully be expanded upon:

1. It would be good to refer to recent research literature to provide more biological context (and useful cross links within the literature). I wonder whether an additional sentence in the introduction outlining the work by Jan Frouz and colleagues (Frouz *et al*. (2019 ^1^), Frouz *et al. *(2015 ^2^)) would be appropriate? 
2. Latitude and longitude are given to five and eight decimal places, respectively. This is an implied precision of approximately 1.1m and 0.7mm. Such precision seems unrealistic for a netted fly, where four decimal places (nearest 10m) seems more likely.
3. I know it is standard DToL methodology, but I would like to see “Manual assembly curation corrected  …” expanded into 2-3 sentences, explaining how curation was done.
4. The absence of an assembled Y chromosome is extremely interesting, and probably warrants a mention in the abstract. I assume the X chromosome is the ‘blob’ that has half the coverage of the rest of the genome? I think the use of coverage to identify potential X and Y chromosome contigs should be mentioned in the methods (if nothing else, this confirms the fly as possessing only one X chromosome).
5. I appreciate that no photo of the sample is available, but I think for context it would be really nice to include a photo of this species, albeit not the actual sample sequenced. I would also like to see a one sentence description (or even a photo!) of the collection habitat.
6. There is no mention of RNAseq, although I believe this is standard for DToL genomes. Is RNAseq data from this sample available? PRJEB45104 suggests that it is, but it is not mentioned here.

Are sufficient details of methods and materials provided to allow replication by others?

Partly

Is the rationale for creating the dataset(s) clearly described?

Yes

Are the datasets clearly presented in a useable and accessible format?

Yes

Are the protocols appropriate and is the work technically sound?

Yes

Reviewer Expertise:

Evolutionary genomics of Drosophila and their pathogens.

I confirm that I have read this submission and believe that I have an appropriate level of expertise to confirm that it is of an acceptable scientific standard.

## References

[1] : Utilization of Dietary Protein in the Litter-Dwelling Larva of Bibio marci (Diptera: Bibionidae). *Eurasian Soil Science* .2019;52(12) : 10.1134/S1064229319120032 1583-1587 10.1134/S1064229319120032

[2] : Major mechanisms contributing to the macrofauna-mediated slow down of litter decomposition. *Soil Biology and Biochemistry* .2015;91: 10.1016/j.soilbio.2015.08.024 23-31 10.1016/j.soilbio.2015.08.024
